# Cardiovascular and renal outcomes of sodium–glucose cotransporter-2 versus dipeptidyl peptidase-4 inhibitors in patients with type 2 diabetes post-PCI: a meta-analysis of 14,511 patients

**DOI:** 10.1186/s13098-025-02080-1

**Published:** 2026-01-09

**Authors:** Ahmed Samy Badran, Mohamed Ibrahim Gbreel, Abdelrahman M. Tawfik, Mahmoud Balata

**Affiliations:** 1 Faculty of Medicine, Ain Shams University, Cairo, Egypt; 2 Faculty of Medicine, October 6 University, Giza, Egypt; 3 Faculty of Medicine, Alexandria University, Alexandria, Egypt; 4 Department of Cardiology, University Hospital Rostock, Ernst- Heydemann-Straße 6, 18057 Rostock, Germany

**Keywords:** Percutaneous coronary intervention, SGLT-2 inhibitors, DPP-4 inhibitors, Type 2 diabetes mellitus, Meta-analysis

## Abstract

**Background:**

Patients with type 2 diabetes mellitus (T2DM) undergoing percutaneous coronary intervention (PCI) are at high risk of adverse cardiovascular and renal outcomes. While both sodium–glucose cotransporter-2 inhibitors (SGLT-2i) and dipeptidyl peptidase-4 inhibitors (DPP-4i) are widely used in this population, direct evidence comparing their long-term efficacy and safety after PCI remains scarce. This meta-analysis aimed to compare cardiovascular and renal outcomes between SGLT-2i and DPP-4i in patients with T2DM post-PCI.

**Methods:**

PubMed, Web of Science, Scopus, and Cochrane CENTRAL were searched through June 2025. Primary outcomes were all-cause mortality, worsening renal function, and heart failure. We included primary studies and assessed the quality of studies using Newcastle Ottawa Scale. RevMan software was used to calculate hazard ratios (HR) estimates and 95% confidence intervals (CI) using the random-effects model.

**Results:**

We analyzed the outcomes between SGLT-2i (*n* = 7,025 patients) and DPP-4i (*n* = 7,459 patients). The mean age was 62.7 years, and 77.4% were males. SGLT-2i significantly reduced all-cause mortality (HR = 0.65; 95% CI: 0.54–0.79; *P* < 0.001) and the risk of worsening renal function (HR = 0.15; 95% CI: 0.09–0.26; *P* < 0.001). They also demonstrated a significant reduction in heart failure events (HR = 0.59; 95% CI: 0.48–0.74; *P* < 0.001). For myocardial infarction, a non-significant trend toward risk reduction with SGLT-2i was observed (HR = 0.85; 95% CI: 0.72–1.02; *P* = 0.08). For cerebrovascular accidents and the need for repeat revascularization (PCI/CABG), no significant difference was observed.

**Conclusion:**

SGLT-2i demonstrates more clinical benefits, and current evidence supports its initiation over DPP-4i in T2DM patients after PCI.

**Supplementary Information:**

The online version contains supplementary material available at 10.1186/s13098-025-02080-1.

## Introduction

Type 2 Diabetes mellitus (T2DM) affects hundreds of millions worldwide and imposes a substantial burden on healthcare systems [1]. It significantly increases the risk of macrovascular and microvascular complications. Cardiovascular disease remains the leading cause of morbidity and mortality among diabetic patients, with coronary artery disease (CAD) remaining the most prevalent cause [2]. Percutaneous coronary intervention (PCI) is considered the cornerstone of revascularization strategies for CAD and is frequently performed in individuals with DM [3]. Despite advancements in interventional cardiology and pharmacotherapy, diabetic patients undergoing PCI have worse angiographic and clinical outcomes. They continue to face a greater risk of adverse cardiovascular events, including in-stent thrombosis, vessel restenosis, recurrent myocardial infarction, and cardiac death, compared to non-diabetics [4]. Thus, effective glycemic control strategies are essential for cardiorenal protection. Additionally, beyond glycemic control, a shift in the management is now towards agents that offer direct cardiovascular and renal protection [5].

Among these therapies, sodium-glucose co-transporter 2 inhibitors (SGLT-2i) and dipeptidyl peptidase-4 inhibitors (DPP-4i) emerged as effective agents for improving T2DM outcomes and reducing complications [6]. SGLT-2i reduces glucose reabsorption in the kidneys [7]. While DPP-4i act by enhancing the endogenous incretin system [8].

Although both classes improve glycemic control, their cardiovascular and renal profiles differ. Major randomized controlled trials (RCTs) like EMPA-REG OUTCOME [9], CANVAS [10], and DECLARE-TIMI 58 [11] have shown that SGLT-2i significantly reduces the risk of major adverse cardiovascular events (MACE), chronic renal failure progression, and mortality in patients with T2DM [12]. These cardioprotective benefits are likely due to a combination of hemodynamic, metabolic, and anti-inflammatory effects of these agents [13]. On the other hand, DPP-4i trials such as SAVOR-TIMI 53 [14] and EXAMINE [15] have generally indicated cardiovascular safety of DPP-4i, their cardioprotective benefits remain uncertain. Nevertheless, DPP-4i are widely used due to their favorable tolerability and low risk of hypoglycemia [16].

Given the distinct cardiovascular and renal profiles of SGLT-2i and DPP-4i, there is growing interest in clarifying their comparative effects in the post-PCI setting among patients with T2DM. Patients with T2DM undergoing PCI constitute a particularly high-risk subgroup [17]. A direct comparison between these two drug classes is clinically relevant, as both are commonly prescribed as second-line glucose-lowering agents. In real-world practice, clinicians managing post-PCI patients with T2DM may face the challenge of managing post-PCI patients who are often poly-medicated, a high-risk subgroup with multiple comorbidities, cardiovascular risk, and safety trade-offs when selecting glucose-lowering therapy [18]. Notably, up to 90% of PCI patients are discharged on five or more medications, and higher medication counts have been independently associated with an increased long-term risk of major bleeding [19, 20]. Previous analyses have largely focused on broader diabetic or cardiovascular populations rather than this specific post-PCI subgroup [12, 21]. For example, Min et al. [22] compared the two classes as an add-on to insulin therapy but focused primarily on glycemic and metabolic outcomes and did not assess cardiovascular endpoints. Similarly, Giugliano et al. [21] evaluated cardiorenal outcomes, yet their analysis did not isolate post-PCI patients as a distinct subgroup.

A comprehensive synthesis of evidence specifically addressing their impact on clinical outcomes in diabetic patients after PCI is limited. This systematic review and meta-analysis aimed to compare the effects of starting SGLT-2i versus DPP-4i regarding cardiovascular and renal outcomes in patients with T2DM after PCI.

## Methods

This study was registered with the International Prospective Register of Systematic Reviews (PROSPERO) under registration number (CRD420251077885). We followed the approaches for conducting the current study based on the Cochrane Handbook of Systematic Reviews on Interventions [23]. The study was reported in accordance with the Preferred Reporting Items for Systematic Reviews and Meta-Analyses (PRISMA) statement guidelines [24].

### Search strategy

We conducted comprehensive research of the PubMed, Scopus, Web of Science, and CENTRAL (Cochrane Central Register of Controlled Trials) databases. The search was performed without language or date restrictions, from database inception up to 10 June 2025. We used combinations of Medical Subject Headings (MeSH) and free-text terms relevant to SGLT-2i, DPP-4i, and PCI. Boolean operators were applied to structure the search strategy. The detailed search strings used in each database are provided in Supplementary Table 1**.** Additionally, we manually screened reference lists of relevant articles and included studies to identify additional eligible records.

### Eligibility criteria

Studies were included according to the following criteria: (1) Population: Adult patients with T2DM who underwent PCI; (2) Intervention: SGLT-2i; (3) Comparison: DPP-4i; (4) Outcomes: cardiovascular, cerebrovascular, and renal outcomes; (5) Study Design: comparative studies, whether interventional or observational studies were eligible for inclusion. On the other hand, the following exclusion criteria were applied: (1) non-comparative studies not comparing SGLT-2i versus DPP-4i; (2) studies without cardiovascular, renal, or hospitalization outcome data; (3) conference abstracts without primary data; (4) editorials or letters to editor; (5) Case reports or case series; (6) review articles.

### Screening and study selection

Two independent authors (A.S.B. and A.T.) screened all titles and abstracts against the inclusion criteria. Eligible full-text articles were subsequently reviewed for final inclusion. Discrepancies between reviewers were resolved through discussion or, if necessary, by a third reviewer (M.I.G.). Duplicate entries were removed prior to screening using EndNote software [25], and the entire selection process was documented using a PRISMA flow diagram.

### Data extraction and endpoints

Two authors (A.S.B. and A.T.) independently extracted data from included studies into a standardized spreadsheet. Extracted data included: (1) study summary characteristics: author, publication year, country, design, sample size, study duration, duration of follow-up, inclusion criteria and outcomes assessed; (2) population baseline characteristics: age, sex, BMI, smoking, diabetes duration, comorbidities, and glucose lowering drugs used; (3) intervention and comparator details: specific agents used, doses, duration; (4) primary endpoints: all-cause mortality, myocardial infarction, cerebrovascular events (stroke), and heart failure; and (5) secondary endpoints: worsening of renal function and need for repeat revascularization. Any disagreements in data extraction were resolved by consensus or, if necessary, by a third reviewer (M.I.G.).

### Quality assessment

We assessed the risk of bias in included studies using the Newcastle-Ottawa Scale (NOS) for non-randomized studies [26]. Two independent reviewers (A.S.B. and A.T.) conducted the assessment. NOS evaluates studies based on three domains: selection of study groups, comparability of groups, and ascertainment of outcome. Studies were classified as low, moderate, or high risk of bias based on their total scores.

### Statistical analysis

All statistical analyses were performed using Review Manager (RevMan) version 5.4. For dichotomous outcomes, such as cardiovascular events, we pooled hazard ratios (HRs) with 95% confidence intervals (CIs), which account for both the number and timing of events throughout each study’s period. To minimize confounding, when available, adjusted propensity score–matched (PSM) models effect estimates were extracted and pooled. The inverse variance (IV) statistical method was used for pooling effect estimates across studies. We employed a random-effects model to account for potential clinical and methodological heterogeneity across studies. Heterogeneity among studies was quantified using the I² statistic, with values of more than 50% considered high heterogeneity [27]. A Cochran’s Q test was also performed, with a P-value < 0.10 considered statistically significant for heterogeneity. Sensitivity analyses were conducted by excluding one study at a time (leave-one-out approach) to evaluate the robustness of the pooled estimates.

## Results

### Literature search

**Fig. 1:**
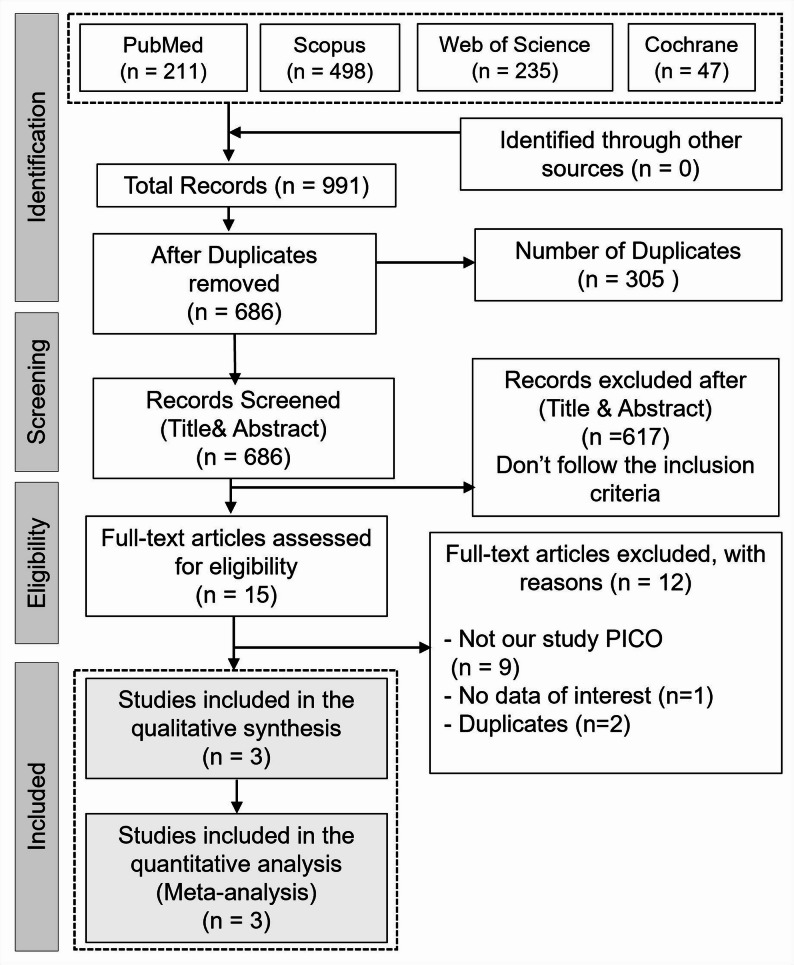
PRISMA flow diagram of the literature search

The electronic databases search yielded 991 records. After duplicates removal, 686 records underwent title and abstract screening. Fifteen studies were evaluated in full-text screening. Ultimately, three studies met the inclusion criteria and were analyzed. Figure 1.

### Characteristics of the included studies

Three studies [28–30] with a total sample of 14,511 patients were analyzed. All studies were observational cohort. The SGLT-2i group 7025, compared to 7459 patients in the DPP-4i group. The mean age of patients ranged from 59.11 to 66.12. 77.4% were males. Metformin was the most concomitantly used anti-diabetic agent among all patients. Tables 1 and 2 provide a detailed summary of the patients and included studies.

**Table 1:** Summary of the included studies

Study ID	Design	Setting	Sample size	Study duration (Year)	Follow-up duration	Population definition	Outcomes assessed	
Kim et al. [30]	Retrospective cohort PSM	South Korea	5512	January 2014– December 2019	5 years	Patients with a history of PCI and new use of SGLT-2 inhibitors or DPP-4i were defined as initiation without prior use of either drug class.	Repeat revascularization (PCI or CABG), Acute MI, stroke, heart failure, all-cause mortality, and end-stage renal disease (ESRD).	
Lee et al. [29]	Retrospective cohort PSM	Tiwan	8220	May 2016– December 2019	4.5 years	Patients with T2DM following PCI who received their first prescription of either an SGLT2-i or DPP-4i were included.	Ischemic stroke, Acute MI, heart failure hospitalization, coronary revascularization (PCI or CABG), composite renal outcomes, lower limb amputation, and all-cause mortality.	
Lyu et al. [28]	Prospective multicenter cohort	South Korea	779	June 2016– June 2020	1 years	Patients with Acute MI, undergoing PCI, and coexisting T2DM are receiving one regimen of MET + SGLT2i or MET + DPP-4i.	MACE, including all-cause mortality, non-fatal MI, revascularization, stroke, and rehospitalization. Also, each MACE component and cardiac/non-cardiac death.	

**Table 2:** Baseline characteristics of the included studies

Study ID	Arms	Sample	Age (year), mean (SD)	Gender, male (%)	BMI (Kg/m2), mean (SD)	Smoking, *N* (%)	Diabetes duration (years), mean (SD)	Comorbidities, *N* (%)			Glucose-lowering agents, number (%)					
								HTN	Dyslipidemia	Prior CVS	Insulin	Sulfonylurea	Metformin	Meglitinides	Thiazolidinedione	α-Glucosidase inhibitor
**Kim et al.** [30]	SGLT-2i	2756	62.72 (9.83)	2105 (76.38)	26.58 (3.47)	673 (24.42)	6.4 (5.41)	2451 (88.93)	2684 (97.39)	NA	459 (16.65)	1058 (38.39)	1871 (67.89)	33 (1.2)	275 (9.98)	149 (5.41)
	DPP-4i	2756	62.75 (10.32)	2121 (76.96)	26.48 (3.52)	699 (25.36)	6.28 (5.28)	2441 (88.57)	2685 (97.42)	NA	456 (16.55)	1055 (38.28)	1876 (68.07)	29 (1.05)	267 (9.69)	136 (4.93)
**Lee et al.** [29]	SGLT-2i	4110	61.7 (11.3)	3236 (78.73)	NA	NA	NA	3201 (77.88)	3402 (82.77)	279 (6.79)	1730 (42.09)	2332 (56.74)	2564 (62.38)	235 (5.72)	585 (14.23)	475 (11.56)
	DPP-4i	4110	62.3 (10.8)	3265 (79.44)	NA	NA	NA	3198 (77.81)	3416 (83.11)	296 (7.20)	1710 (41.61)	2396 (58.30)	2604 (63.36)	215 (5.23)	572 (13.92)	468 (11.39)
**Lyu et al.** [28]	SGLT-2i	186	59.11 (11.52)	150 (80.7)	76 (43.4) *	110 (61.1)	98 (73.7) ‡	113 (60.8)	48 (25.8)	12 (6.5)	0 (0)	0 (0)	186 (100)	0 (0)	0 (0)	0 (0)
	DPP-4i	593	66.12 (10.86	422 (71.2)	221 (40.0) *	278 (48.7)	259 (65.4) ‡	395 (66.7)	134 (22.6)	56 (9.5)	0 (0)	0 (0)	593 (100)	0 (0)	0 (0)	0 (0)

### Quality assessment

Using the Newcastle-Ottawa scale (NOS), all included studies were rated as of good quality. Supplementary Table 2 provides detailed judgments across various domains.

### Outcomes

#### All-cause mortality

**Fig. 2:**
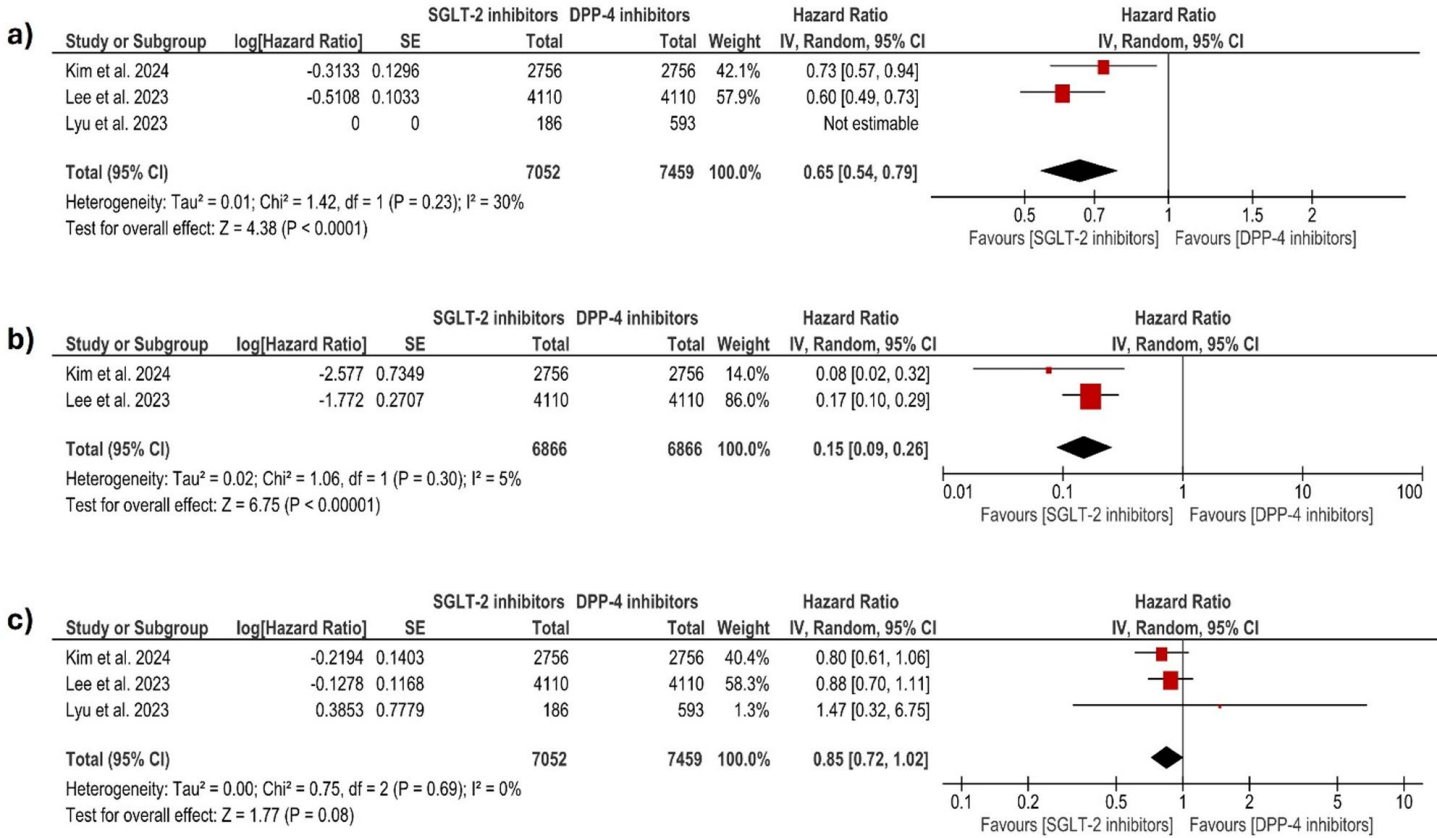
Forest plot comparing (**a**: all-cause death, **b**: worsening renal function, and **c**: myocardial infarction) between SGLT-2 inhibitors and DPP-4 inhibitors in patients with type 2 diabetes after PCI. The size of each square represents the study weight, and the horizontal lines denote 95% CIs. The diamond indicates the pooled estimate. SGLT-2: sodium–glucose cotransporter-2; DPP-4: dipeptidyl peptidase-4; HR: hazard ratio; CI: confidence interval; SE: standard error; IV: inverse variance; PCI: percutaneous coronary intervention

Compared to DPP-4i, SGLT-2i were associated with better outcomes in all-cause mortality. The pooled analysis, which was calculated from Kim and Lee et al. [29, 30] with follow-up durations of 4.5 and 5 years, respectively, demonstrated a statistically significant reduction in the risk of death among patients treated with SGLT-2i (HR = 0.65; 95% CI [0.54–0.79]; *P* < 0.0001). The analysis showed low heterogeneity (I² = 30%, *p* = 0.23). Figure 2a.

#### Worsening of renal function

The outcome of worsening renal function was assessed in two studies comparing SGLT-2i (6866 patients) to DPP-4i (6866 patients). The pooled analysis demonstrated a significantly lower risk of renal function deterioration in patients treated with SGLT-2i (HR = 0.15; 95% CI [0.09–0.26]; *P* < 0.0001). The analysis showed low heterogeneity (I² = 5%, *P* = 0.3). Figure 2b.

#### Myocardial infarction

Three studies reported the outcome of myocardial infarction with 7052 patients in the SGLT-2i group and 7459 patients in the DPP-4i group. The meta-analysis yielded an HR of 0.85 95% CI: [0.72–1.02]; *P* = 0.08, indicating a non-statistically significant trend reduction in myocardial infarction risk with SGLT-2i compared to DPP-4i. The studies were homogenous (I² = 0%, *P* = 0.83). Figure 2c.

#### Cerebrovascular events

**Fig. 3:**
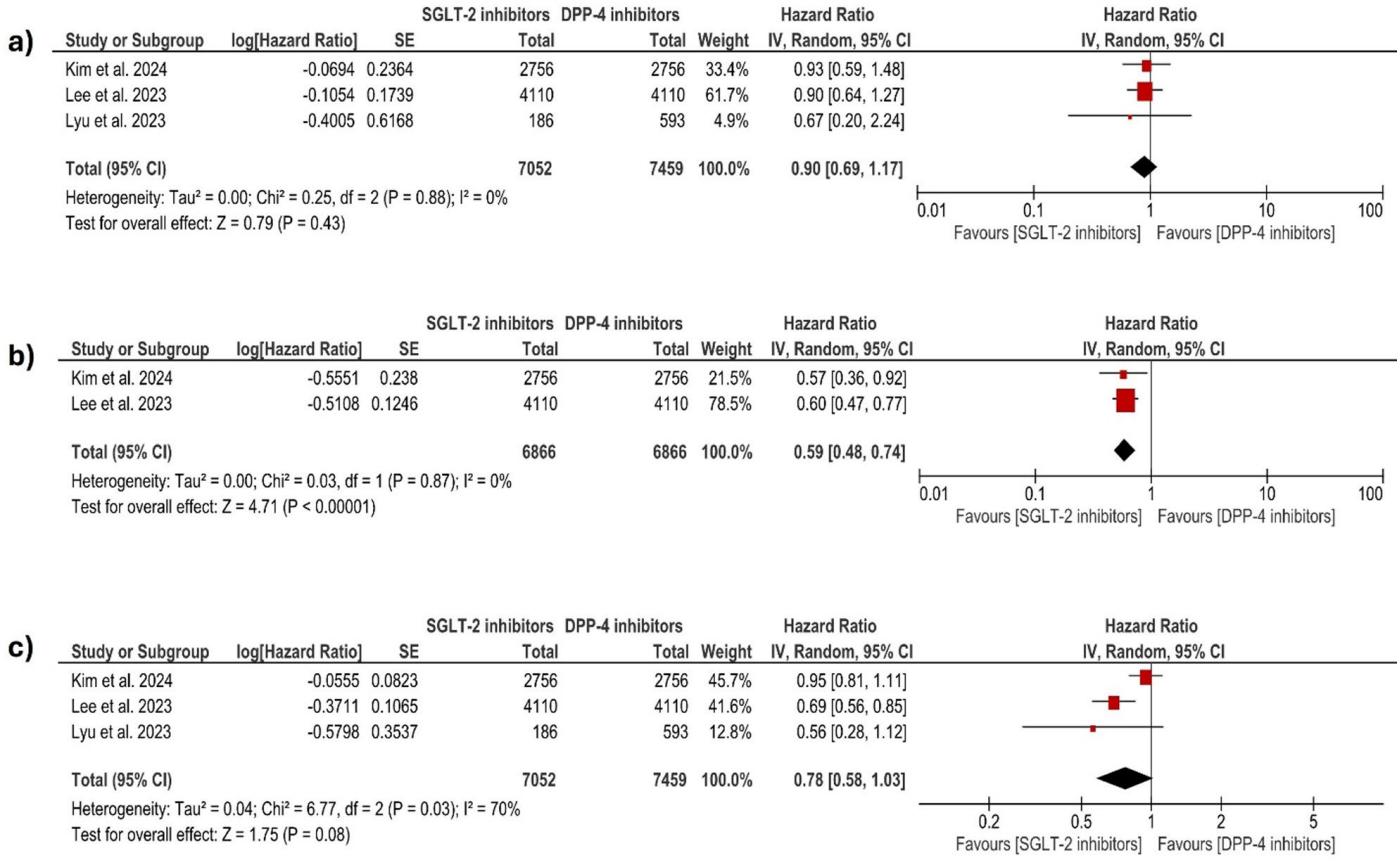
Forest plot comparing (**a**: cerebrovascular accidents, **b**: heart failure, and **c**: repeat revascularization) between SGLT-2 inhibitors and DPP-4 inhibitors in patients with type 2 diabetes after PCI. The size of each square represents the study weight, and the horizontal lines denote 95% CIs. The diamond indicates the pooled estimate. SGLT-2: sodium–glucose cotransporter-2; DPP-4: dipeptidyl peptidase-4; HR: hazard ratio; CI: confidence interval; SE: standard error; IV: inverse variance; PCI: percutaneous coronary intervention

The meta-analysis compares the risk of cerebrovascular accidents (e.g., stroke) among patients treated with SGLT-2i (7052 patients) and those receiving DPP-4i (7459 patients) across three studies. The pooled HR is 0.90; 95% CI: [0.69–1.17]; *p* = 0.43, showing no significant difference between SGLT-2i and DPP-4i. The analysis showed low heterogeneity (I² = 0%, *p* = 0.88). Figure 3a.

#### Heart failure

The meta-analysis evaluated the risk for the patient to present with heart failure events among patients using SGLT-2i (6866 patients) versus DPP-4i (6866 patients). The pooled analysis yields an overall HR of 0.59; 95% CI: [0.48–0.74]; *p* < 0.001, indicating a significant reduction in heart failure risk compared to DPP-4i. The analysis showed low heterogeneity (I² = 0%, *p* = 0.87). Figure 3b.

#### Need for repeat revascularization (PCI or CABG)

The risk of repeat revascularization (PCI or CABG) between patients treated with SGLT-2i and those on DPP-4i. The pooled HR is 0.78; 95% CI: [0.58–1.03]; *p* = 0.08. indicating a non-statistically significant trend in the SGLT-2i group to reduce the revascularization incidence. Heterogeneity was moderate (I² = 70%, *p* = 0.03). Figure 3c.

## Discussion

This meta-analysis directly compares SGLT-2i with DPP-4i in T2DM patients undergoing PCI, a uniquely high-risk cohort. Prior meta-analyses have evaluated these agents in broader diabetic or cardiovascular populations but have not isolated post-PCI patients.

Our findings underscore several key differences and similarities in the cardiorenal and mortality outcomes between SGLT-2i and DPP-4i in this context. Specifically, SGLT-2i demonstrated a statistically significant reduction in all-cause mortality, worsening of renal function, and heart failure events. In contrast, for myocardial infarction, cerebrovascular events, and repeated revascularization, our analysis indicated no statistically significant difference between SGLT-2i and DPP-4i.

We found a statistically significant reduction in all-cause mortality with SGLT-2i, consistent with individual reports by Kim et al. and Lee et al. [29, 30]. Although Lyu et al. [28] did not achieve statistical significance—likely reflecting their shorter one-year follow-up—their data trended similarly. The mechanisms underlying this mortality benefit likely encompass both direct cardiovascular effects and non-cardiovascular improvements. SGLT-2i have been shown to reduce cardiovascular death and hospitalization for heart failure involving a broader T2DM population [31]. Additionally, SGLT-2i has been shown to enhance myocardial energetics by shifting cardiac metabolism toward more efficient fuel use, especially through increased ketone body production [32]. The hemodynamic effects of SGLT-2i, including blood pressure reduction and intravascular volume optimization (reduction in cardiac preload and afterload), may contribute to reduced cardiovascular strain [33]. Beyond these cardiovascular mechanisms, SGLT-2i may also reduce mortality through non-cardiovascular pathways such as decreased risk of inflammation and oxidative stress, improved metabolic control, and direct renal effects that alleviate cardiorenal syndrome [34, 35].

Our analysis showed no significant differences between SGLT-2i and DPP-4i in the risk of myocardial infarction or cerebrovascular accidents. These neutral findings are consistent with the known mechanisms of action of SGLT-2i, which primarily exert hemodynamic and metabolic effects, such as reductions in preload, afterload, and plasma volume, rather than direct modulation of atherosclerotic plaque or thrombosis. This aligns with prior repoted neutral effects of SGLT-2i on atherosclerotic events [36, 37]. Collectively, these observations underscore that SGLT-2i should complement, rather than replace, established anti-atherosclerotic therapies such as statins and antiplatelet agents in post-PCI patients. Despite the fact that SGLT-2i didn’t lessen the occurrence of myocardial infarction, a previous study has suggested that it may play a protective role regarding the prognosis of these patients [38].

The meta-analysis revealed a significant reduction in heart failure events with SGLT-2i. This is a consistent finding across all included studies and aligns with the well-established benefits of SGLT-2i in heart failure [31, 39]. The mechanisms underlying this benefit likely include several unique pharmacological actions of SGLT-2i. By inhibiting sodium-glucose co-transport in the proximal tubule, these agents promote natriuresis and osmotic diuresis, leading to reductions in both preload and afterload [40]. Importantly, they achieve these hemodynamic benefits without activating neurohormonal systems such as the renin-angiotensin-aldosterone system (RAAS). Additionally, SGLT-2i have been shown to inhibit myocardial Na⁺/H⁺ exchange, potentially reducing intracellular sodium and calcium overload in cardiomyocytes [41]. These combined effects may explain why Lyu et al. observed significant improvements in left ventricular ejection fraction (LVEF) with SGLT-2i despite not reporting heart failure outcomes specifically [28]. These benefits are particularly critical in post-PCI diabetic patients who are at an elevated risk of developing or worsening heart failure [42].

Regarding the risk of worsening renal function, SGLT-2i showed a favorable outcome than DPP-4i. This finding indicates a renoprotective effect of SGLT-2i, which likely originates from their ability to reduce intraglomerular pressure through tubuloglomerular feedback mechanisms. By inhibiting sodium and glucose reabsorption in the proximal tubule, they increase distal sodium delivery to the macula, which constricts the afferent arteriole and then reduces glomerular hyperfiltration [43]. This mechanism may be particularly important in diabetic patients (who are at high risk for progressive kidney disease) and also post-PCI (who might be at high risk for contrast-induced nephropathy). Additionally, post‑PCI T2DM patients on SGLT‑2i experienced significantly fewer contrast‑related AKI events than those on DPP‑4i [44]. These renoprotective effects of SGLT-2i align with the results of landmark trials like CREDENCE, DAPA-CKD, and EMPA-KIDNEY [45–47]. Yet, according to Chen et al., these benefits may wane in advanced CKD. Collectively, this may encourage early initiation of SGLT2i for at-risk diabetic patients. The reduction in renal deterioration observed in our analysis (HR = 0.15) is substantial but should be interpreted with caution. This effect may be influenced by variability in the definition endpoints and follow-up duration. Consequently, the finding should be considered preliminary and warrant confirmation in large, standardized prospective studies.

The need for repeat revascularization showed a non-significant trend toward reduction with SGLT-2i, though with substantial heterogeneity. This heterogeneity likely reflects differences in study populations and revascularization strategies. Lee et al. reported a significant reduction (HR 0.69), while Kim et al. found no difference (HR 0.98). While SGLT-2i are known for their heart failure and renal benefits, their direct anti-atherosclerotic effects are less consistently demonstrated in broader populations. Further studies with longer follow-up periods and standardized definitions of revascularization events would be beneficial to clarify this relationship.

Our findings complement and extend prior evidence while differing in methodology and population focus. Giugliano et al. [21] conducted a broad synthesis of cardiovascular and renal outcome trials reporting effects of GLP-1 receptor agonists, SGLT-2i, and DPP-4i in patients with or without T2DM, and reported that SGLT-2i significantly reduced cardiovascular death (RR = 0.88) and all-cause mortality (RR = 0.87) compared with DPP-4 inhibitors. However, their analysis did not isolate post-PCI patients as a distinct clinical subgroup, limiting direct applicability to secondary prevention following PCI. Similarly, Ansari et al. [48] addressed a related comparison, but the inclusion criteria, outcomes, and follow-up considerations differed from those applied in our analysis. Ansari et al. [48] also compared SGLT-2i and controls following PCI. Our analysis extends their findings in several important ways. First, we focused exclusively on direct head-to-head comparisons between SGLT-2i and DPP-4i, employing pooled HRs to appropriately account for differences in follow-up duration across studies. Second, our work incorporated additional outcomes, including renal deterioration and myocardial infarction, offering a more comprehensive assessment of cardiorenal risk. Notably, our results for stroke differ from those reported by Ansari et al., who observed a significant reduction with SGLT-2i use (RR = 0.77; 95% CI: 0.62–0.96; *p* < 0.01), whereas our pooled HR (0.90; 95% CI: 0.69–1.17; *p* = 0.43) showed no significant difference between drug classes. This discrepancy may suggest that SGLT-2i offers limited benefit for atherosclerotic outcomes in the post-PCI setting and warrants a standalone strategy for lipid control, or that both classes provide comparable vascular protection within this specific population.

Regarding glycemic targets, Lyu et al. reported HbA1c changes, showing a greater reduction with SGLT-2i compared with DPP-4i (ΔHbA1c: −1.15 ± 2.00 vs. −0.65 ± 1.90; *P* = 0.04). In contrast, Kim et al. and Lee et al. acknowledged the absence of HbA1c data. Previous literature indicates that generally both drug classes exert comparable glucose-lowering effects, with mean reductions of approximately 0.4–0.7% [49]. Therefore, the consistent cardiovascular and renal benefits observed with SGLT-2i across studies are unlikely to be solely explained by differences in glycemic control. These findings suggest that SGLT-2i may confer additional cardioprotective and renoprotective effects independent of glucose lowering, through mechanisms such as improved hemodynamic stability, reduced cardiac preload and afterload, and attenuation of renal hyperfiltration. This aligns with the recommendations favoring SGLT-2i use in high-risk patients with T2DM irrespective of baseline HbA1c levels [50, 51].

It is also essential to recognize that the prognosis of PCI patients is influenced by a multifactorial interaction of metabolic, inflammatory, demographic, and procedural factors. PCI outcomes are influenced by patient- and lesion-specific characteristics, each contributing to clinical risk. Patient-related factors such as age, sex, renal dysfunction, insulin resistance, and systemic inflammation have demonstrated significant impact on the prognosis. Insulin resistance is associated with poorer cardiovascular outcomes, including higher rates of MACEs, MI, and mortality, even in non-diabetic populations [52–54]. Lesion complexity further modulates risk, as chronic total occlusion, multivessel disease, and bifurcation lesions are strongly associated with procedural difficulty, increased likelihood of restenosis, and adverse event rates. Procedural determinants, including the number/length of implanted stents and the degree of revascularization completeness, also contribute to long-term prognosis [53, 54]. Sex-based differences add another layer. Women with acute coronary syndrome undergoing PCI demonstrate higher risks of MACEs and bleeding compared with men, a pattern attributed in part to older age and higher comorbidity burden at presentation. In contrast, women with stable coronary artery disease may experience better adjusted long-term survival following PCI [55, 56]. Ultimately, a holistic and individualized therapeutic strategy that considers this wide array of variables is essential for optimizing outcomes for patients undergoing PCI.

### Clinical implications

The findings of this meta-analysis have meaningful clinical implications. SGLT-2i were associated with lower risks of mortality, heart failure, and renal events. These findings are consistent with the established cardiorenal benefits of SGLT-2i demonstrated in large RCTs. Current recommendations advocate for SGLT-2i use high cardiovascular or renal risk [50, 51]. Within the context of post-PCI diabetic patients, a population underrepresented in RCTs, our results suggest that SGLT-2i may be the preferred therapeutic option over DPP-4i, particularly among those with pre-existing cardiorenal vulnerability. The neutral effects on atherosclerotic outcomes underscore the importance of integrating SGLT-2i therapy within a broader cardiovascular risk reduction strategy including optimized lipid control, antithrombotic, and blood pressure management. Clinicians should remain mindful of class-specific adverse effects, such as genital infections and volume depletion, particularly in elderly or renally impaired patients. In contrast, DPP-4i remain alternatives for those with contraindications or intolerance to SGLT-2i.

### Limitations

Several limitations warrant consideration. First, the meta-analysis was based on three studies. Although these studies collectively included a large sample size and were methodologically robust, the small number of available datasets restricts statistical power, external validity, and assessment of publication bias. While observational studies provide valuable real-world evidence, they are inherently susceptible to confounding by unmeasured variables, despite efforts such as propensity score matching; residual confounding could still influence the observed associations. Additionally, observational studies introduce a potential bias in comparative outcomes between agents regarding adherence to antidiabetic therapy. Second, the follow-up periods in the studies varied, with Lyu et al. having a relatively short one-year follow-up, which might not be sufficient to capture the full story for some outcomes. Third, the high heterogeneity limits the certainty of the pooled estimate for repeat revascularization outcome, opposite to all other outcomes, which showed low heterogeneity. Fourth, the populations in the included studies were predominantly Asian (Korean and Taiwanese), which might limit the generalizability of our findings to other ethnic or racial groups. Fifth, information on glycemic control was not consistently available across studies.

#### Future recommendations

Future research should include large-scale randomized trials with extended follow-up durations, and standardized definitions for the outcomes’ definition e, g., the renal deterioration. It should also evaluate SGLT-2i versus DPP-4i and other antidiabetic agents such as GLP-1 receptor agonists. Comparative and combination studies are particularly warranted to elucidate whether targeting complementary pathophysiologic mechanisms may yield additive cardiovascular and renal benefits. GLP-1 receptor agonists have demonstrated reductions in atherosclerotic cardiovascular events and body weight, particularly among patients with established ASCVD, whereas SGLT-2i have shown superior effects in reducing heart failure incidence and preserving renal function. Investigating the potential synergistic effects of combining SGLT-2i and GLP-1 receptor agonists may therefore represent an important next step in optimizing outcomes for patients with T2DM undergoing PCI [57, 58]. Research should also explore whether specific SGLT-2i agent classes offer differential benefits and whether combination therapy with DPP-4i might provide additive effects. Moreover, studies should explore the effects of SGLT-2i in diverse populations’ ethnicity beyond the predominantly Asian cohorts included in the current meta-analysis.

## Conclusion

In patients with T2DM after PCI, SGLT2i demonstrate more clinical benefits compared to DPP4i. Specifically, SGLT2i significantly reduces all-cause mortality, hospitalizations for heart failure, and the risk of renal function decline. However, no significant differences were observed between the two drug classes in the incidence of myocardial infarction, stroke, or repeat revascularization. Despite this, the overall risk–benefit profile favors the use of SGLT2i in this high-risk population. These results should be confirmed in future randomized trials.

## Supplementary Information

Supplementary Material 1

## Data Availability

The data that support the findings of this study are available from the corresponding author upon reasonable request.

## Abbreviations

HR: Hazard ratio
CI: Confidence Interval
PRISMA: Preferred Reporting Items for Systematic Reviews and Meta-Analyses
RCTs: Randomized Clinical Trials
DM: Diabetes mellitus
SGLT-2i: Sodium–glucose cotransporter 2 inhibitors
DPP-4i: Dipeptidyl peptidase-4 inhibitors 4 inhibitors
PCI: Percutaneous Coronary Intervention
CABG: Coronary Artery Bypass Graft
ASCVD: Atherosclerotic cardiovascular disease

## Declarations

### Trial registry number

This meta-analysis was registered on PROSPERO. Registration Number: CRD420251077885.
