# Supplementary material for: Cardiovascular and renal outcomes of sodium–glucose cotransporter-2 versus dipeptidyl peptidase-4 inhibitors in patients with type 2 diabetes post-PCI: a meta-analysis of 14,511 patients
Source: Diabetol Metab Syndr. 2026 Jan 9;18:63. doi: 10.1186/s13098-025-02080-1 (PMC12903319; doi:10.1186/s13098-025-02080-1)
Supplement: Supplementary file 1 — Supplementary Material 1 [file 13098_2025_2080_MOESM1_ESM.docx]

**Supplementary Materials**

- **Supplementary Table 1.** Search strategy for each database.
- **Supplementary Table 2.** Quality assessment of the included studies using the Newcastle–Ottawa Scale (NOS).

| **Supplementary Table 1.** Search Strategy for each database. | | |
| --- | --- | --- |
| **Database** | **Strategy** | **Results** |
| **PubMed** | ( ("SGLT2" OR "sodium-glucose cotransporter 2" OR "sodium glucose cotransporter 2" OR "sodium–glucose co-transporter 2" OR SGLT2i OR gliflozin* OR empagliflozin OR dapagliflozin OR canagliflozin OR ertugliflozin OR bexagliflozin OR ipragliflozin OR tofogliflozin OR luseogliflozin OR remogliflozin OR sotagliflozin OR (SGLT2 NEAR/5 inhibitor*)) AND ("DPP4" OR "DPP-4" OR "dipeptidyl peptidase 4" OR "dipeptidyl-peptidase IV" OR DPP4i OR gliptin* OR sitagliptin OR saxagliptin OR linagliptin OR alogliptin OR vildagliptin OR teneligliptin OR anagliptin OR trelagliptin OR omarigliptin OR gemigliptin OR (DPP4 NEAR/5 inhibitor*)) AND ( "percutaneous coronary intervention" OR PCI OR "coronary stent*" OR "coronary angioplasty" OR "PTCA" OR "coronary intervention*" OR "coronary revascularization" OR (coronary NEAR/5 stent*) OR (PCI NEAR/5 outcome*) OR "after PCI" OR "post-PCI" OR "myocardial infarction" OR MI OR "acute myocardial infarction" OR STEMI OR NSTEMI OR "heart attack" OR (myocardial NEAR/5 infarct*) OR (MI NEAR/5 outcome*) ) ) | 211 |
| **Web of Science (WOS)** | TS=( ( "SGLT2" OR "sodium-glucose cotransporter 2" OR "sodium glucose cotransporter 2" OR "sodium–glucose co-transporter 2" OR SGLT2i OR gliflozin* OR empagliflozin OR dapagliflozin OR canagliflozin OR ertugliflozin OR bexagliflozin OR ipragliflozin OR tofogliflozin OR luseogliflozin OR remogliflozin OR sotagliflozin ) AND ( "DPP4" OR "DPP-4" OR "dipeptidyl peptidase 4" OR "dipeptidyl-peptidase IV" OR DPP4i OR gliptin* OR sitagliptin OR saxagliptin OR linagliptin OR alogliptin OR vildagliptin OR teneligliptin OR anagliptin OR trelagliptin OR omarigliptin OR gemigliptin ) AND ( "percutaneous coronary intervention" OR PCI OR "coronary stent*" OR "coronary angioplasty" OR PTCA OR "coronary intervention*" OR "coronary revascularization" OR (coronary NEAR/5 stent*) OR (PCI NEAR/5 outcome*) OR "post-PCI" OR "after PCI" OR "myocardial infarction" OR MI OR "acute myocardial infarction" OR STEMI OR NSTEMI OR "heart attack" ) ) | 235 |
| **Scopus** | TITLE-ABS-KEY ( ( ( "SGLT2" OR "sodium-glucose cotransporter 2" OR "sodium glucose cotransporter 2" OR "sodium–glucose co-transporter 2" OR sglt2i OR gliflozin* OR empagliflozin OR dapagliflozin OR canagliflozin OR ertugliflozin OR bexagliflozin OR ipragliflozin OR tofogliflozin OR luseogliflozin OR remogliflozin OR sotagliflozin ) AND ( "DPP4" OR "DPP-4" OR "dipeptidyl peptidase 4" OR "dipeptidyl-peptidase IV" OR dpp4i OR gliptin* OR sitagliptin OR saxagliptin OR linagliptin OR alogliptin OR vildagliptin OR teneligliptin OR anagliptin OR trelagliptin OR omarigliptin OR gemigliptin ) AND ( "percutaneous coronary intervention" OR pci OR "coronary stent*" OR "coronary angioplasty" OR ptca OR "coronary intervention*" OR "coronary revascularization" OR ( coronary AND near/5 AND stent* ) OR ( pci AND near/5 AND outcome* ) OR "post-PCI" OR "after PCI" OR "myocardial infarction" OR mi OR "acute myocardial infarction" OR stemi OR nstemi OR "heart attack" ) ) ) | 498 |
| **Cochrane Library** | ( ( "SGLT2" OR "sodium-glucose cotransporter 2" OR "sodium glucose cotransporter 2" OR "sodium–glucose co-transporter 2" OR SGLT2i OR gliflozin* OR empagliflozin OR dapagliflozin OR canagliflozin OR ertugliflozin OR bexagliflozin OR ipragliflozin OR tofogliflozin OR luseogliflozin OR remogliflozin OR sotagliflozin ) AND ( "DPP4" OR "DPP-4" OR "dipeptidyl peptidase 4" OR "dipeptidyl-peptidase IV" OR DPP4i OR gliptin* OR sitagliptin OR saxagliptin OR linagliptin OR alogliptin OR vildagliptin OR teneligliptin OR anagliptin OR trelagliptin OR omarigliptin OR gemigliptin ) AND ( "percutaneous coronary intervention" OR PCI OR "coronary stent*" OR "coronary angioplasty" OR PTCA OR "coronary intervention*" OR "coronary revascularization" OR (coronary NEAR/5 stent*) OR (PCI NEAR/5 outcome*) OR "post-PCI" OR "after PCI" OR "myocardial infarction" OR MI OR "acute myocardial infarction" OR STEMI OR NSTEMI OR "heart attack" ) ) in Title Abstract Keyword | 47 |
| **Total results** | | 991 |

| **Supplementary Table 2.** Quality assessment of the included studies using the Newcastle Ottawa scale (NOS). | | | | | | | | | |  |
| --- | --- | --- | --- | --- | --- | --- | --- | --- | --- | --- |
| **study ID** | **Selection** | | | | **Comparability** | **Outcome** | | | **Overall Score (out of 9)** |  |
|  | Representativeness of the exposed cohort (score:★) | Selection of the non exposed cohort (score:★) | Ascertainment of exposure(score:★) | Demonstration that outcome of interest was not present at start of study(score:★) | Comparability of cohorts on the basis of the design or analysis(score :★★) | Assessment of outcome(score:★) | Was follow-up long enough for outcomes to occur(Maximum:★) | Adequacy of follow up of cohorts(Maximum:★) |  |  |
|  |  |  |  |  |  |  |  |  |  |  |
| Kim et al. 2024 | ★ | ★ | ★ | ★ | ★★ | ★ | ★ | ★ | 9 |  |
| Lee et al. 2023 | ★ | ★ | ★ | ★ | ★★ | ★ | ★ | ★ | 9 |  |
| Lyu et al. 2023 | ★ | ★ | ★ | ★ | ★ | ★ | ★ | ★ | 8 |  |
